# Effect of High Glucose-Induced Oxidative Stress on Paraoxonase 2 Expression and Activity in Caco-2 Cells

**DOI:** 10.3390/cells8121616

**Published:** 2019-12-11

**Authors:** Camilla Morresi, Laura Cianfruglia, Davide Sartini, Monia Cecati, Stefania Fumarola, Monica Emanuelli, Tatiana Armeni, Gianna Ferretti, Tiziana Bacchetti

**Affiliations:** 1 Department of Life and Environmental Sciences, Polytechnic University of Marche, Via Brecce Bianche, 60131 Ancona, Italy; m.morres@libero.it (C.M.); moniacecati@gmail.com (M.C.); t.bacchetti@staff.univpm.it (T.B.); 2 Department of Clinical Sciences, Polytechnic University of Marche, Via Brecce Bianche, 60131 Ancona, Italy; l.cianfruglia@staff.univpm.it (L.C.); d.sartini@staff.univpm.it (D.S.); s.fumarola@pm.univpm.it (S.F.); m.emanuelli@staff.univpm.it (M.E.)

**Keywords:** advanced glycation end products, hyperglycemia, intestinal cells, oxidative stress, paraoxonase2

## Abstract

(1) Background: Hyperglycemia leads to several biochemical and physiological consequences, such as the generation of advanced glycation end products (AGEs) and reactive oxygen species (ROS), which are involved in the development of several human diseases. Intestinal cells are continuously exposed to pro-oxidants and lipid peroxidation products from ingested foods, and also to glyco-oxidative damage. It has been reported that free radical generation may be linked to the development of inflammation-related gastrointestinal diseases. (2) Methods: The effects of high glucose (HG) treatment (50 mM) were assessed in terms of free radical production, lipid peroxidation, and AGEs formation. Furthermore, the expression and the antiapoptotic and antioxidant activity of the paraoxonase-2 (PON2) enzyme in intestinal cells has been investigated. (3) Results: Caco-2 cells treated with media supplied with high glucose (HG) (50 mM) showed, with respect to physiological glucose concentration (25 mM), an increase in ROS production, lipid peroxidation, and AGEs formation. Moreover, a lower PON2 expression and activity in HG-treated cells was related to activation of the apoptotic pathways. (4) Conclusions: Our results demonstrated that high glucose concentrations triggered glyco-oxidative stress in intestinal cells; the downregulation of PON2 could result in a higher oxidative stress and might contribute to intestinal dysfunction.

## 1. Introduction

Intestinal cells are highly vulnerable to oxidative damage due to the exposure to luminal oxidants and lipid peroxidation products from ingested foods [1,2,3,4]. It has been reported that reactive oxygen species (ROS) generation and alterations of antioxidant defense mechanisms may be linked to the development of gastrointestinal diseases such as inflammatory bowel disease (IBD) [1,2,3,4,5,6].

At a molecular level, it has been observed that high levels of reactive oxygen (ROS) and nitrogen (RNS) species contribute to cell and tissue dysfunction and damage. ROS can behave also as signaling molecules that regulate a wide variety of physiological functions: inducing programmed cell death or necrosis; inducing or suppressing the expression of many genes, and activating cell signaling cascades [7,8,9]. ROS can also lead to the disruption of cell signaling transduction pathways. The translocation of transcription factors to the nucleus to the antioxidant response element (ARE) induces the production of several endogenous antioxidant enzymes [9]. Some pathways modulated by ROS and oxidative stress activate NF-κB and expression of proinflammatory cytokines [8,10,11].

Diabetes is associated with oxidative stress and is considered a risk factor for chronic degenerative diseases. Moreover, gastrointestinal complications of diabetes mellitus have been described [12,13]. Among dietary factors able to trigger oxidative stress in intestinal cells, there are consumption of high glycemic index foods and intake of advanced glycation products [14,15,16]. At the molecular level, there is evidence that hyperglycemia causes oxidative stress due to hydroxyl radicals generated by autoxidation of glucose [17,18]. This reaction represents an early stage for development of long-term complications, associated with high glucose exposition, and leads to the formation of advanced glycation end products (AGEs) and/or glyco-oxidation of intracellular and extracellular proteins [19]. In particular, elevated levels of intracellular AGEs lead to protein cross-linking and aggregation resulting in an alteration in cell signaling, which causes cell damage and death. These alterations are implicated in diabetes and various chronic pathologies [6,20,21].

Among antioxidant enzymes, the paraoxonase (PON) family, which includes paraoxonase 1 (PON1), paraoxonase 2 (PON2), and paraoxonase 3 (PON3), has been widely studied. PONs are calcium-dependent enzymes that have physiopathological roles, mainly investigated in cardiovascular and neurodegenerative diseases [22,23,24,25,26]. PON2 is expressed in intestinal epithelial cells and exerts antioxidant properties [27,28,29,30,31]. The intracellular enzyme is localized in the plasma membrane, endoplasmic reticulum, nuclear envelope, and the inner mitochondrial membrane. The protective effect against intracellular ROS formation and lipid peroxidation exerted by PON2 has been confirmed in different experimental models and in Caco-2 cells in culture [27,31]. PON2 silencing predisposes cells to lipid peroxidation and to the development of an exacerbated inflammatory response in intestinal epithelial cells [27]. On the contrary, the addition of purified PON2 to permeabilized intestinal Caco-2 cells decreases lipid peroxidation after induction of oxidative stress [30]. At the molecular level, the protective effects of PON2 in different models (human endothelial EA.hy 926 cells, HeLa cell line, and the livers and hearts obtained from C57BL/6J mice), are in part mediated by the role of PON2 in mitochondrial function [32,33]. In detail, in these cell models, PON2 is found associated with respiratory complex III and directly binds to coenzyme Q_10_, an essential constituent of the electron transfer chains (ETC), in a calcium-dependent manner. Furthermore, PON2 prevents mitochondrial superoxide (O_2_^•–^) formation and decreases its release by both mitochondrial complex I and complex III at the inner mitochondrial membrane [33]. The antiapoptotic properties of PON2 have been related to its modulatory role of cytochrome c release from mitochondria and caspase activation [33,34]. Other authors have suggested that the antioxidant and antiapoptotic roles of PON2 could be also exerted in other subcellular compartments, such as the ER [35,36].

Recent studies have shown that chronic glucose stress induces downregulation of PON2 mRNA expression with consequent decreases in enzymatic activity in macrophages and monocytes, and contributes to an increased production of reactive oxygen species (ROS) [37]. The effect of high glucose on intestinal cells and PON2 expression and activity has not been previously studied. The principal aim of the study was to investigate the effect of high glucose chronic exposure on free radical production, AGEs formation, and expression of PON2 using Caco-2 cells as a model of intestinal cells. We also tested the effect on antioxidant defenses, caspase activation, and apoptosis.

## 2. Materials and Methods

### 2.1. Reagents and Antibodies

All cell culture reagents were obtained by Euroclone (Euroclone, Italy). All chemical reagents were obtained by Sigma Aldrich (Sigma, St Louis, MO, USA). Carboxy-H2DCFDA (C400) was supplied by Invitrogen (Invitrogen, Carlsbad, CA, USA). Human colon epithelial cells Caco-2 (ATCC^®^HTB-37™) were purchased from the American Type Culture Collection (Rockville, MD, USA). Total RNA Isolation System and M-MLV reverse transcriptase were purchased from Promega (Madison, WI, USA). RNeasy Micro Kit was obtained from Qiagen (Hilden, Germany). All reagents for Real-Time PCR were purchased from Bio-Rad Laboratories (Hercules, CA, USA). Rabbit polyclonal PON2 (SAB2700275), rabbit polyclonal β-actin (A2066), and goat polyclonal anti-AGE (AB9890) antibodies were purchased from Sigma-Aldrich (St. Louis, MO, USA). Rabbit monoclonal cleaved caspase-3 (#9664), mouse monoclonal caspase-8 (#9746), mouse polyclonal phospho-p53 (#9284), mouse polyclonal p53 (#2524), rabbit monoclonal mitofusin-2(#124773), rabbit monoclonal TOM20 (#42406), and rabbit monoclonal TNFα (#3707) antibodies were purchased from Cell Signaling Technologies (Leiden, Netherlands). Rabbit polyclonal caspase-9 antibody (ab25758) was purchased from Abcam (England and Wales UK).

### 2.2. Cell Culture and Incubation with Glucose

Caco-2 cells (passages 10–26, ATCC) were cultured in Dulbecco’s minimal essential medium (DMEM) supplemented with 10% (*v*/*v*) heat-inactivated fetal bovine serum (FBS), 2 mM glutamine, 100 U/mL penicillin, 100 µg/mL streptomycin, and 10 mM nonessential amminoacids at 37 °C in a humidified atmosphere containing 5% (*v/v*) CO_2_. Cells were treated with isotonic media containing physiological (25 mM) or high glucose (HG) (50 mM) concentrations [16] for 1 week. Medium was replaced two times per week.

### 2.3. Cell Extracts 

Cells were trypsinized and centrifuged at 1200× *g* for 10 min. Pellets were washed twice in phosphate-buffered saline (PBS). The extracts were obtained by resuspending cellular pellets with extraction buffer containing sodium phosphate buffer pH 6.8, protease inhibitors (2.08 mM 4-(2-Aminoethyl) benzene sulfonyl fluoride hydrochloride, 1.6 mM aprotinin, 0.08 mM bestatin, 0.03 mM E-64, 0.04 mM leupeptin, 0.3 mM pepstatin A, and 0.5% NP40 detergent. All procedures were carried out at 4 °C. Supernatants were recovered and used to evaluate protein content [38] and other biochemical parameters (fluorescent AGEs levels, total antioxidant activity, Western blot analysis, and activity of antioxidant enzymes).

### 2.4. Western Blot Analysis 

Cell extracts containing 50 μg protein were subjected to 12.5% sodium dodecyl sulfate polyacrylamide gel electrophoresis and transferred to polyvinylidene fluoride (PVDF) membranes. After regular blocking and washing, the membranes were incubated with specific primary antibodies overnight at 4 °C. For the expression of molecules the products involved in the regulation of the apoptosis pathway were rabbit monoclonal cleaved caspase-3 antibody, mouse monoclonal caspase-8, rabbit polyclonal caspase-9 antibody, mouse polyclonal phospho-p53 antibody, mouse polyclonal p53. For the expression of molecules involved in the regulation of mitochondria rabbit monoclonal mitofusin-2 and rabbit monoclonal TOM20 were used. For the expression of molecules involved in the inflammation rabbit monoclonal cells, TNFα was used. For the analysis of paraoxonase-2, rabbit polyclonal PON2 was used. For the determination of glycolaldehyde-modified proteins (GA-modified proteins), goat polyclonal anti-AGE antibody was used. β-actin was used as loading control. Donkey anti-goat, goat anti-mouse, and goat anti-rabbit secondary antibodies HRP (horseradish peroxidase) were used in accordance with the manufacturer’s instructions. Protein bands were developed by the enhanced SuperSignal West Femto Maximum Sensitivity Substrate (Thermo Fisher Scientific, Waltham, MA, USA). The chemiluminescent signal was acquired using ChemiDoc XRS+ System (Bio-Rad Laboratories, Hercules, CA, USA) and analyzed by using the Image J software (Version 1.50i, National Institute of Health, Bethesda, MD, USA).

### 2.5. Quantitative Real-Time PCR

Each frozen pellet of Caco-2 cells, treated in different experimental conditions, were homogenized in a lysis buffer. Total RNA was extracted through the SV total RNA Isolation System (Promega, Madison, WI, USA) and was isolated using the RNeasy Micro Kit (Qiagen, Hilden, Germany), according to the manufacturer’s instructions. Total RNA was reverse transcribed in a total volume of 25 μL for 60 min at 37 °C with M-MLV reverse transcriptase (Promega, Madison, WI, USA), using random primers.

To examine PON2 gene expression quantitatively, we performed real-time PCR analyses using the CFX96 Real-Time PCR Detection System (Bio-Rad Laboratories, Hercules, CA, USA). cDNA, generated as previously described, was used as the template. To avoid false positive results caused by amplification of contaminating genomic DNA in the cDNA preparation, all primers were selected to flank an intron. PCR efficiency was tested for both primer pairs and found to be close to 1. The primers used were (forward) 5′-TCGTGTATGACCCGAACAATCC-3′ and (reverse) 5′-AACTGTAGTCACTGTAGGCTTCTC-3′ for PON2and (forward) 5′-TCCTTCCTGGGCATGGAGT-3′ and (reverse) 5′-AGCACTGTGTTGGCGTACAG-3′ for β-actin. Genes were run in duplicate for 40 cycles at 95 °C for 30 s and 58 °C for 30 s, using SsoFastEvaGreenSupermix (Bio-Rad Laboratories, Hercules, CA, USA). All samples were tested in triplicate with the reference gene β-actin for data normalization. Direct detection of PCR products was monitored by measuring the fluorescence produced by Eva Green dye binding to double strand DNA after every cycle. mRNA levels were normalized to the mRNA levels of the housekeeping gene ß-actin.

### 2.6. Intracellular ROS Levels 

Intracellular ROS levels were detected by flow cytometry using H_2_DCFDA (C400) as probe. Cells were trypsinized, washed twice with cold PBS, and suspended at a final concentration of 0.5 × 10^6^ cell/mL in prewarmed PBS containing 10 µM probe. After incubation for 30 min in the dark at 37 °C, cells were washed twice in PBS and stained with 10 µg/mL propidium iodide (PI). Fluorescence of labelled cells was measured on Guava easy Cyteflow cytometer (Merck Millipore, Darmstadt, Germany) using an excitation wavelength of 488 nm. Emissions were recorded using the green channel for carboxy-DCF and the red channel for propidium iodide [39]. The cells permeable to PI were excluded from the cell population considered for the ROS production to avoid false negatives. The data acquired were analyzed by the FCS Express Program (De Novo Software, CA, USA).

### 2.7. Cell Lipid Peroxidation

Lipid peroxidation products were quantified by measuring thiobarbituric acid reactive substances (TBARS). One mL of 20% (*w*/*v*) trichloroacetic acid containing 0.8% (*w*/*v*) thiobarbituric acid (TBA) was added to each culture dish. The cells were scratched off and the suspensions were transferred to glass centrifuge tubes and boiled for 45 min. After centrifugation the absorbance of the supernatant at 535 nm was determined. Using the molar extinction coefficient of the (malondialdehyde)MDA–TBA complex of 1.49 × 10^5^ M^−1^ cm^−1^, the amount of TBARS was expressed as nmol MDA equivalents formed per mg cell protein [40].

### 2.8. Cell Total Antioxidant Activity

The antioxidant activity of Caco-2 cells treated in different experimental conditions was performed by oxygen radical absorbance capacity (ORAC)assay [41]. Antioxidant activity was expressed as mM Trolox Equivalents (TE)/10^6^ cells.

### 2.9. Evaluation of Fluorescent Advanced Glycation End Products (AGEs)

Levels of fluorescent AGEs were detected in cell extracts by evaluating intrinsic fluorescence of AGEs (340 nm/420 nm as excitation and emission wavelengths) (Synergy microplate reader, BioTek Instruments, Inc.). Results were expressed by fluorescence intensity per mg cell proteins [42].

### 2.10. MTT Test 

Cells viability was analyzed by 3-(4,5-dimethylthiazol-2-yl)-2,5-diphenyltetrazolium bromide tetrazolium reduction (MTT) assay [43]. Briefly, CaCo-2 cells. after 6 days of treatment, were seeded at a density of 5 × 10^4^ cells/well in to a 96-well plate at different conditions and incubated at 37 °C in an atmosphere of 5% CO_2_. After plating overnight, 100 μL of MTT solution (5 mg/mL) was added to each well. After 2 h, the incubation buffer was removed and the blue MTT–formazan product was extracted with DMSO (dimethyl sulfoxide). Supernatants were collected in a 96-well plate and the absorbance was measured at 540 nm (Microplate Rader).

### 2.11. Apoptosis Analysis

Apoptosis was analyzed by cytometric analysis, using FITC Annexin V Apoptosis Detection Kit (Biolegend, San Diego, CA, USA) according to the manufacturer’s instructions. Briefly, cells were trypsinized, washed twice with cold PBS, and 10^6^ cell/mL were resuspended in 1X binding annexin V-FITC (0.25 µg/mL) and propidium iodide (PI) (1 µg/mL) were added to cell suspension and incubated, protected from light, for 10 min at room temperature. Samples were analyzed using Guava easyCyteflow cytometer (Merck Millipore, Darmstadt, Germany). For each sample, 5000 events were acquired [44]. Annexin V-FITC is detected as a green fluorescence and propidium iodide is detected as a red fluorescence. Early apoptosis is defined by annexin V+/PI− staining, late apoptosis is defined by annexin V+/PI+ staining, and necrosis is defined by annexin V−/PI+ staining).

### 2.12. Determination of Caspase-3 and Caspase-8 Activity

Caspase-3 and caspase-8 activities were determined using the Caspase-3/CPP32 and Caspase-8/FLICE Colorimetric Assay Kits (Biovision, Milpitas, CA, USA), respectively, in cells treated in different experimental conditions. Cells were trypsinized, centrifuged at 500× *g* for 5 min at 4 °C, resuspended in 50 μL lysis buffer provided by each kit, and incubated on ice for 10 min. Cell lysates were then centrifuged at 10,000× *g* for 1 min at 4 °C and supernatant was used for further analysis. After determination of protein concentration, 150 µg protein extract were transferred to a 96-plate well and incubated with 50 μL 2× reaction buffer containing 10 mmol/L DTT and 4 mmol/L DEVD-p-nitroanilide substrate (for caspase-3) or IETD-p-nitroanilide substrate (for caspase-8), followed by 1 h incubation at 37 °C. Cells treated without substrates represented negative control samples. Optical density (OD) for each specimen was determined at 405 nm using a microtiter plate reader.

### 2.13. Enzymatic Activity Assays

For enzymatic assay, cells were trypsinized and washed twice in PBS. Protein extract assay was obtained by resuspending pellet in Triton X-100 RIPA Buffer (50 mM Tris-HCl pH 8.0, 150 mM NaCl, 2 mM EDTA, 0.2% Triton X-100) and proteinase inhibitor. After 40 min incubation on ice, cell lysate was centrifuged at 12,000× *g* for 15 min at 4 °C. Supernatants were then recovered and total protein concentration was determined by the Bradford protein assay.

#### 2.13.1. Paroxonase 2 (PON2) Activity

Paroxonase2 (PON2) activity in cellular extracts was measured using 5-thiobutylbutyrolactone (TBBL) a synthetic substrate gently provided by Dr. Tawfik, from the Weizmann Institute of Science (Rehovot, Israel) [45]. The reaction mix contained 22 mM Tris-HCl pH 8.0, 1.0 mM CaCl_2_, 0.2 mM TBBL, 0.5 mM 5,5′-dithiobis 2-nitrobenzoic acid (DTNB = Ellman’s reagent), and 0.5% DMSO. Stock solutions were 200 mM TBBL in acetonitrile and 100 mM DTNB in DMSO. Reactions were initiated by the addition of DTNB and TBBL (5 min later) to cellular extract (50 µg protein). Plates were read at 412 nm (at 49 s intervals between reads) in a plate reader. One unit of lactonase activity is to 1 μmol of TBBL hydrolyzed/mL/min.

#### 2.13.2. Glutathione Reductase (GR) Activity

Glutathione reductase (GR) activity was analyzed by the method described by Carlberg and Mannervik [46], which measures the decrease in absorbance at 340 nm due to NADPH oxidation during GSSG reduction. The assay was performed in 100 mM sodium phosphate (pH 7.0), 100 µM NADPH, and 1 mM GSSG; GR activity was calculated using an extinction coefficient (εmM) for NADPH of −6.22 mM^−1^ × cm^−1^ and expressed as µmol of NADP^+^ per min per mg of proteins.

#### 2.13.3. Glutathione Peroxidase (GPX) Activity

Glutathione peroxidase (GPX) activity was measured using cumene hydroperoxide as substrate. This activity was assayed in a coupled enzyme system, where NADPH is consumed by glutathione reductase to convert the formed GSSG to its reduced form (GSH). The decrease of absorbance of NADPH was monitored at 340 nm (εmM = −6.22 mM^−1^ × cm^−1^) using 0.8 mM cumene hydroperoxide in 100 mM potassium phosphate pH 7.5, 1 mM EDTA, 2 mM GSH, 0.15 mM NADPH, and 1 unit of GR.

#### 2.13.4. Catalase (CAT) Activity

Catalase (CAT) activity was determined by using 12 mM hydrogen peroxide (H_2_O_2_), as substrate, in 100 mM potassium phosphate pH 7.0 and measuring the decrease in absorbance at 240 nm (εmM = 0.04 mM^−1^ × cm^−1^) due to the consumption of H_2_O_2_.

### 2.14. Statistical Analysis 

The data from cell experiments are representative of three independent experiments and the data are shown as mean ± SD. For comparison between the two groups, Student’s t-test was applied, and differences were considered to be significantly different if *p* < 0.05 (Origin, OriginLab Corporation).

## 3. Results

### 3.1. Effect of High Glucose (HG) Treatment on ROS Levels and AGEs in Caco-2 Cells

**Figure 1:**
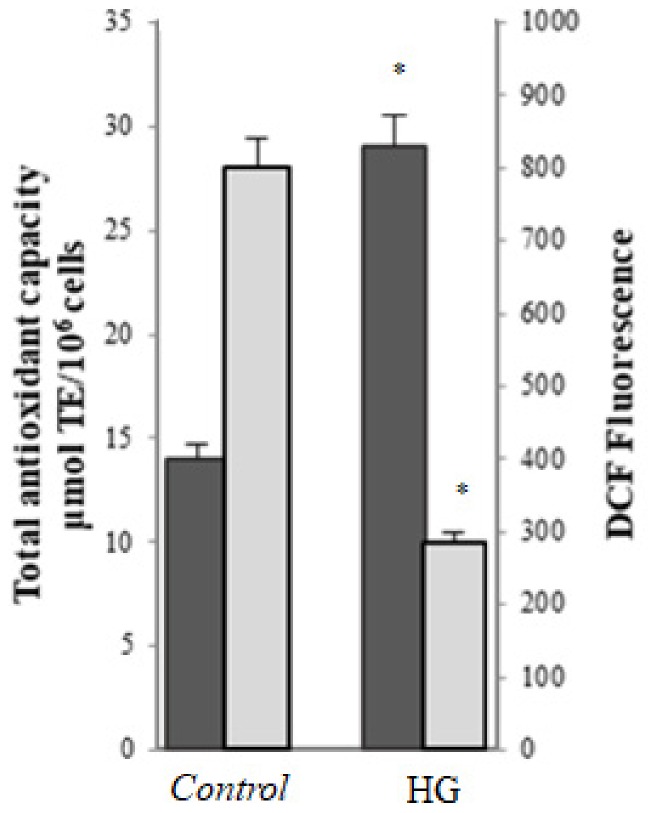
Intracellular ROS production evaluated using DCF fluorescence (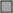) and total antioxidant activity (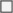) in control (25 mM glucose) or high glucose (HG) (50 mM glucose) cells. Data are presented as means ± SD of three independent experiments carried out in triplicate (*n* = 9). * *p* ≤ 0.05 vs. control cells.

**Figure 2:**
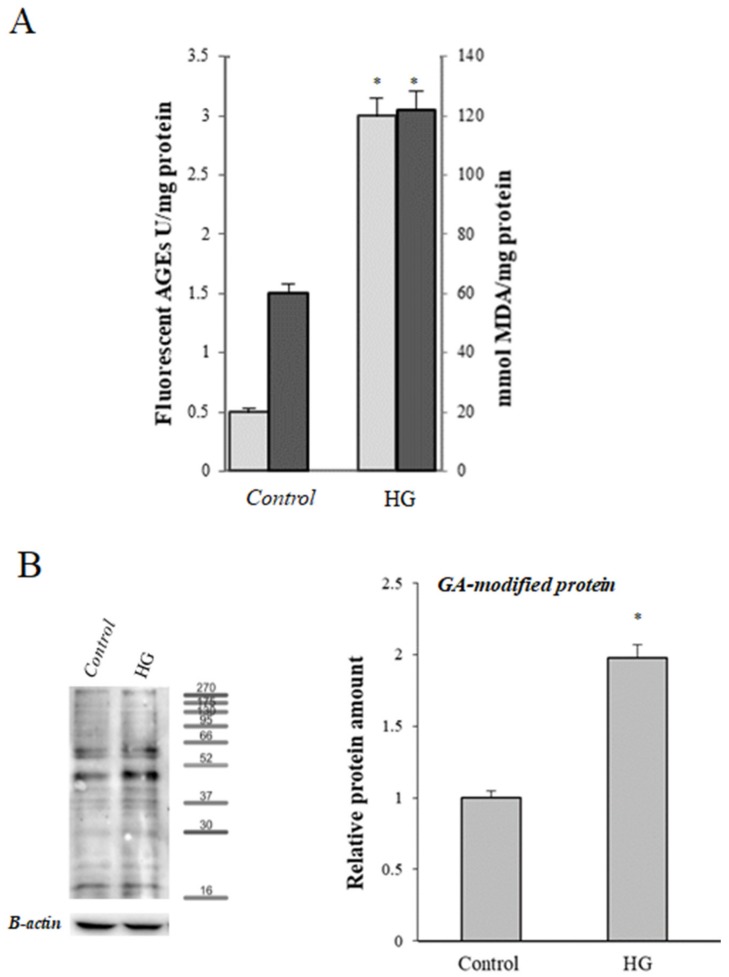
Protein and lipid modification induced by high glucose treatment. (**A**) Levels of total fluorescent AGEs (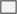) and levels of malondialdehyde (MDA) (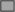) in control (25 mM glucose) or high glucose (HG) (50 mM glucose) cells. (**B**) Representative Western blot and relative densitometric analysis of total GA-modified proteins in control or high glucose (HG) cells. Data are normalized on β-actin. Values reported are expressed as mean ± standard deviation of three independent experiments carried out in triplicate (* *p* < 0.05 vs. control cells).

Intracellular ROS levels, evaluated using carboxy-H_2_DCFDA, were significantly increased after treatment with high glucose (HG-cells) compared with control cells (*p* < 0.001); moreover, a significant decrease of total intracellular antioxidant activity was observed in HG cells compared with control (*p* < 0.001) (Figure 1). Levels of lipid peroxidation products (TBARS levels) and fluorescent AGEs were significantly higher in HG-treated cells in comparison to control cells (Figure 2A). To further investigate the formation of AGEs in cells treated in different conditions, the levels of glycolaldehyde-modified proteins (GA-modified proteins) were evaluated using Western blot analysis. As shown in Figure 2B, compared with the control group, the HG cells had more GA-modified proteins.

### 3.2. Effect of High Glucose (HG) Treatment on PON2 Expression and Activity

**Figure 3:**
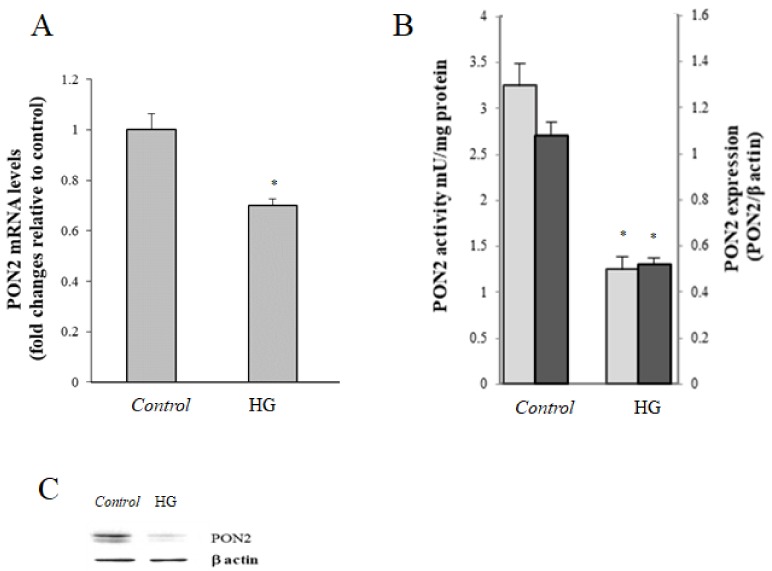
Effect of HG treatment on PON2. (**A**) PON2 mRNA levels evaluated by real-time PCR in control (25 mM glucose) or high glucose (HG) (50 mM glucose) cells. (**B**) Protein PON2 levels (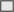) and activity (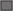) in control (25 mM glucose) or high glucose (HG) (50 mM glucose) cells; data are normalized on β-actin. (**C**) Representative Western blot of PON2 expression. Values reported are expressed as mean ± standard deviation (* *p* ≤ 0.05 vs. control cells).

Results obtained from real-time PCR analyses performed on intestinal cells treated in different experimental conditions showed that PON2 mRNA levels were significantly lower in HG cells with respect to cells incubated in physiological conditions, with a percentage decrease of about 50% (Figure 3A). To confirm these results, PON2 protein levels and activity were investigated. Consistent with the results of real-time PCR, both PON2 protein and activity showed a marked decrease in HG-treated cells compared to the control (Figure 3B,C).

### 3.3. Effect of High Glucose (HG) Treatment on Caspases and Apoptosis

**Figure 4:**
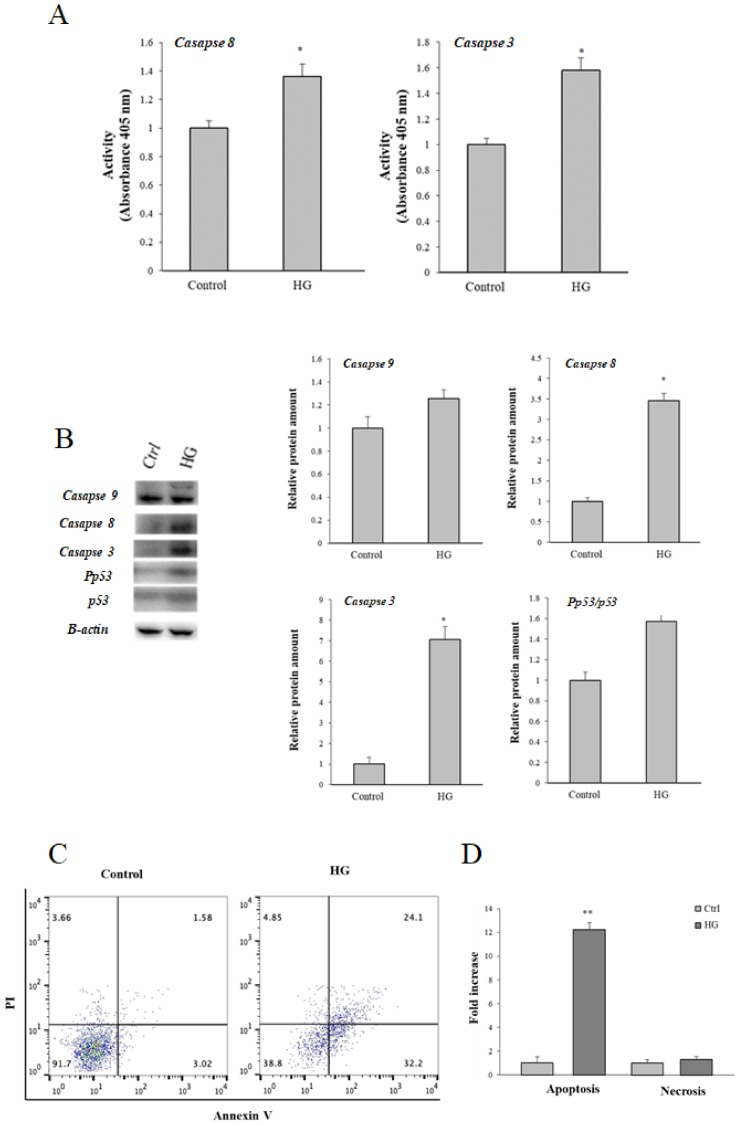
Effects of HG treatment on apoptosis. (**A**) Activity of caspase 8 and caspase 3. (**B**) Representative Western blot and relative densitometric analysis of proteins in control (25 mM glucose) or high glucose (HG) (50 mM glucose) cells, and Pp53/p53 ratio. All results are normalized on β-actin (* *p* ≤ 0.05 vs control cells). (**C**) Representative cytograms from three independent experiments are shown for control (25 mM glucose) and high glucose (HG) (50 mM glucose) cells. (**D**) Apoptosis and necrosis quantified as a fold increase in HG cells with respect to the control. Total apoptosis was calculated by considering early apoptosis (lower right) and late apoptosis (upper right). Data are the mean of at least three experiments ± standard deviation (** *p* ≤ 0.001 vs. control cells).

To investigate the physio-pathological relevance of the modifications of PON2, we investigated whether high glucose treatment triggers caspase cascade activation and apoptosis. As summarized in Figure 4A, significantly higher activity of both caspase-8 and caspase-3 were observed in HG-treated cells compared to control cells. Moreover, higher levels of caspase-8, caspase-9, and effector caspase-3 were observed in cells treated with HG (Figure 4B).

The cell apoptosis rate was detected by flow cytometry after staining with annexin V/propidium iodide (PI). Cells treated with 50 mM glucose exhibited a higher apoptosis rate compared with control cells (Figure 4C,D).

### 3.4. Effect of High Glucose (HG)-Treatment on p53

We analyzed the expression of p53 and its phosphorylated form (Pp53). After normalization and comparison to the relative value of the control, we observed an increase in Pp53 expression in cells after treatment with high glucose concentration (Figure 4B).

### 3.5. Effect of High Glucose (HG) Treatment on Mitochondria

**Figure 5:**
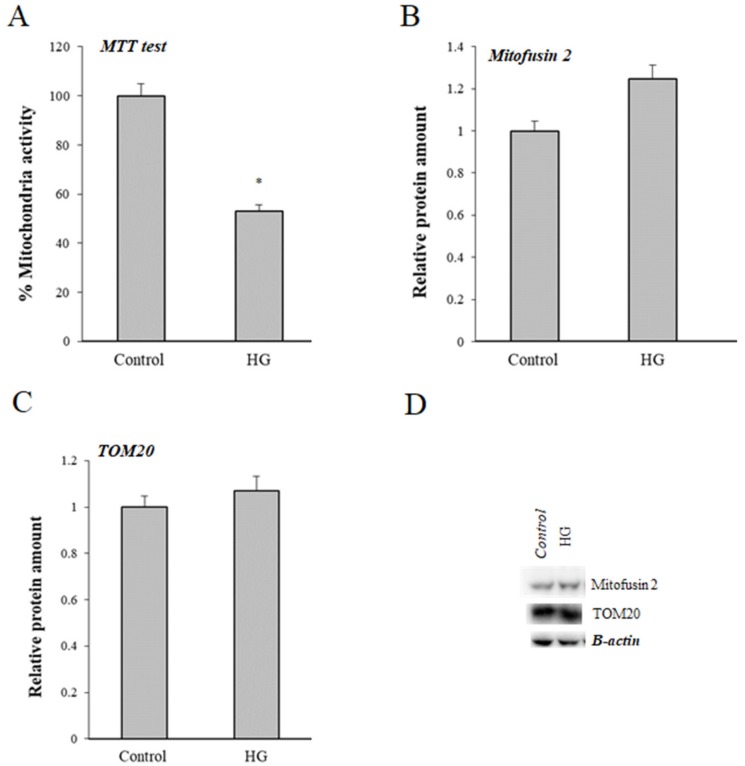
Effects of HG treatment on mitochondria. MTT assay (**A**); protein levels of Mitofusin2 (Mfn2) (**B**); translocase of outer membrane, TOM20 (**C**); representative western blot (**D**), in control (25 mM glucose) or high glucose (HG) (50 mM glucose) cells. All results are normalized on β-actin (* *p* ≤ 0.05 vs. control cells).

Using MTT assay, a decrease of about 40% of formation of MTT formazan was observed in HG-treated cells (Figure 5A). To further investigate whether mitochondria are involved in the mechanisms of glyco-oxidation on intestinal cells, we evaluated expression of two biochemical mitochondrial markers: Mitofusin 2 and TOM20 in control cells and HG-treated cells. As shown in Figure 5B, expression of both markers was increased, although the differences were not statistically significant.

### 3.6. Effect of High Glucose (HG) Treatment on Activity of Glutathione Peroxidase (GPX), Glutathione Reductase (GR), and Catalase (CAT)

**Figure 6:**
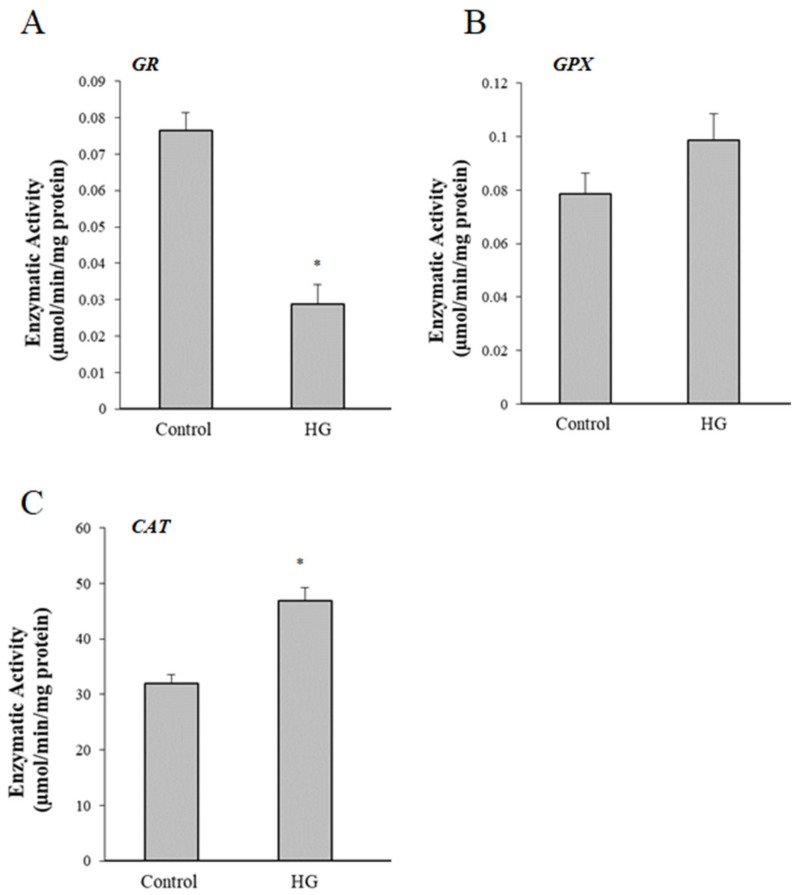
Effects of high glucose treatment on antioxidant enzymes. Enzymatic activity of glutathione reductase (GR) (**A**), glutathione peroxidase (GPX) (**B**), and catalase (CAT) (**C**) in control (25 mM glucose) or high glucose (HG) (50 mM glucose) cells. Results are reported as mean values ± standard deviation (S.D.) of six independent experiments. Statistical analysis was performed from treated vs. untreated cells. * *p* ≤ 0.01 vs control cells.

The activity of other endogenous antioxidant enzymes was analyzed to determine whether the decrease of PON2 in HG-treated cells was associated to alterations of oxidative balance. The activity of the glutathione peroxidase (GPX), glutathione reductase (GR), and catalase (CAT) were evaluated in controls and HG-treated cells. Data showed a significant decrease in glutathione-dependent enzyme GST and GR, while nonsignificant alterations in GPX were found (Figure 6A,B). Catalase activity was significantly increased after HG treatment (Figure 6C).

### 3.7. Inflammatory Response

**Figure 7:**
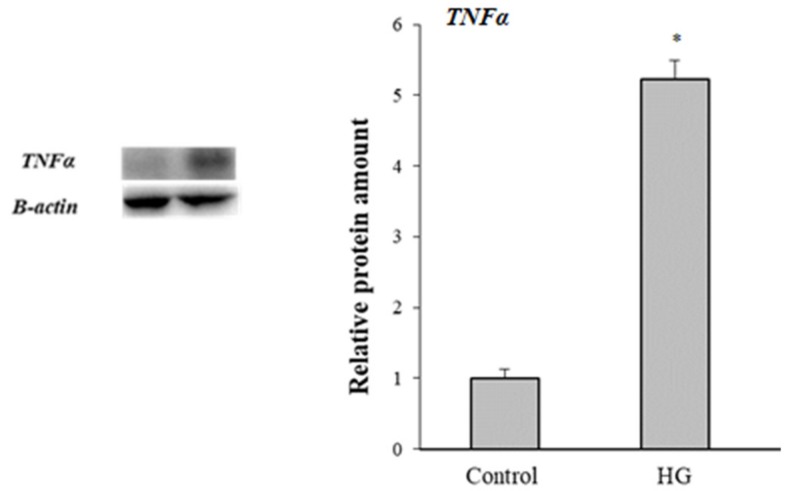
Effects of high glucose treatment on TNFαlevels. Representative Western blot and relative densitometric analysis of protein TNFα levels in control (25 mM glucose) or high glucose (HG) (50 mM glucose) cells. Data are normalized on β-actin. Values reported are expressed as mean ± standard deviation (* *p* ≤ 0.05 *vs* control cells).

Since inflammation and glyco-oxidative stress are closely linked phenomena, we analyzed the modulation of TNF-α levels. As shown in Figure 7, high glucose treatment stimulated the expression of TNF-α in Caco-2 cells.

## 4. Discussion

The enzyme PON2 is widely expressed through the digestive tracts [27,28]. In human intestines, PON2 is associated with nuclei, mitochondria, lysosomes, and microsomes [30]. Using Caco-2 cells as a model of intestinal cells, it has been demonstrated that the enzyme is affected by oxidative stress and inflammation [27,28]. High glucose levels induce oxidative stress in different cells and a relationship between high glucose, oxidative stress, and AGEs formation has been previously demonstrated [17,18,47]. Higher levels of ROS, lipid peroxidation products, and a decrease of total antioxidant capacity have been observed in our experimental conditions in HG-treated Caco-2 cells. Moreover, HG-treated cells showed a significant increase of AGEs, as demonstrated by the increase of fluorescent AGEs and glycolaldehyde (GA)-modified proteins. These compositional changes confirm that glyco-oxidation was realized in our intestinal cell model.

A decrease in PON2 mRNA level, associated with a lower PON2 protein expression and its lactonase activity, has been observed in HG- treated Caco-2 cells. These data are in agreement with other studies in Caco-2 cells oxidized in different experimental conditions. In fact, a decrease of PON2 has been observed in Caco-2 cells oxidized by iron-ascorbate [27] or by treatment with H_2_O_2_ [48]. PON2 mRNA and protein downregulation under hyperglycemic conditions is supported by previous studies on macrophages and monocytes [37,49]. Moreover, a lower PON2 expression has been observed in liver and heart of hyperglycemic animal models [37,49].

Previous studies have demonstrated that mitochondria are a major source of free radical-related oxidative stress [50]. PON2 modulates mitochondrial function and exerts antiapoptotic properties [32,33,34]. At molecular level, the regulatory role of PON2 in mitochondria is related to its localization on the matrix side of the inner mitochondrial membrane [32,33]. In particular, previous studies have shown that the ability of PON2 to prevent the impact of mitochondrial O_2_^•–^ formation on mitochondrial proapoptotic signaling [34].To investigate the effect of HG treatment on mitochondrial functionality, we evaluated the levels of Mitofusin2 and TOM20. As recently reviewed, mitofusin 2 is involved in several cell pathways, as well as in the pathogenesis of metabolic disorders [51]. TOM20 is a member of the translocase family of the outer mitochondrial membrane and it acts as a general import receptor for mitochondrial proteins and allows movement of proteins through this barrier and into the intermembrane space of the mitochondrion [52]. Expression of mitochondrial mitofusin 2 and TOM20 was not significantly modified in our experimental conditions, however the MTT assay has demonstrated a decrease of about 40% of formation of MTT formazan in HG-treated cells. The reaction is catalyzed by mitochondrial succinate dehydrogenase, hence, the MTT assay is dependent on mitochondrial respiration and indirectly serves to assess the cellular energy capacity. Therefore, we suggest that functioning of mitochondria, could be altered during glyco-oxidative stress of Caco-2 cells. Other hypotheses can be formulated to explain the relationship between PON2 and the higher intracellular ROS formation in HG-treated cells. Of considerable interest is the localization of PON2 in a number of intracellular compartments. Subcellular fractionation analyses revealed the distribution of PON2 in nuclei, mitochondria, lysosomes, and microsomes in the intestine [30]. Studies are underway in our laboratory to address and better understand the role of PON2 in the modulation of oxidative stress and its active participation in the protection of organelle integrity and function during oxidative stress.

The higher expression of caspase 8, caspase 9, and effector caspase 3, and the higher apoptosis rate detected by flow cytometry, demonstrated that apoptosis is also promoted in HG-treated cells. Other authors have demonstrated that high glucose toxicity triggers apoptosis in different cell models through different mechanisms [53,54,55]. Among factors involved in regulation of apoptosis, p53 plays an important role. This factor is implicated in oxidative stress and diabetes-associated complications [55]. Under stress conditions such as chronic hyperglycemia, p53 is activated through post-transcriptional modifications, which appear to influence its participation in cell apoptosis [55,56]. Activated p53 triggers the transcription of several genes involved in regulating oxidative stress. Among p53 target genes, there are antioxidant enzymes such as glutathione peroxidase (GPX) [56]. Post-translational modifications of p53, including phosphorylation, are common ways to activate p53 in response to DNA damage and perturbation of phospholipid homeostasis [57]. The increased phosphorylation of p53 correlates with an increase in reactive oxygen species, a release of cytochrome c, and an increase in the rate of apoptosis [58]. Therefore, we hypothesize that the significant increase of Pp53/p53 ratio in Caco-2 cells incubated with high glucose is related to glyco-oxidative stress and contributes to apoptosis pathways.

Oxidative stress can induce proinflammatory genes, such as TNF-α. The increased expression of TNF-α in our cell model demonstrated that glyco-oxidative stress predisposes an inflammatory response. An increased TNF-α expression and IL-1β have been observed in PON-2-deficient mice treated with lipopolysaccharide (LPS) [59].

The decrease of PON2 lactonase activity under chronic glucose stress could also result from glyco-oxidative modification. The effect of glycation on structure and functions of proteins has been widely investigated. Previous studies have shown that PON enzymes are sensitive to glycation triggered by glucose or methylglyoxal (MGO), which induces loss of biological activity [60,61,62]. Even oxidative stress could contribute to alterations of PON2 activity. Indeed, PON2 contains three cysteine groups: the disulfide bond Cys 42–Cys 353, the free cysteine at position 284, and an additional free cysteine at position 311 [63]. Loss of PON2 enzymatic activity could also result in cysteine oxidation, leading to disulfide bond formation between cysteine residues in different PON2 molecules [63,64]. Additional investigation is needed to elucidate the underlying mechanism of regulation for PON2 gene expression during glyco-oxidative stress.

The decrease of activity of glutathione reductase and the increase of the activity of catalase, involved in modulation of H_2_O_2_ levels, confirm alteration of antioxidant/oxidant balance in HG-treated cells.

**Figure 8:**
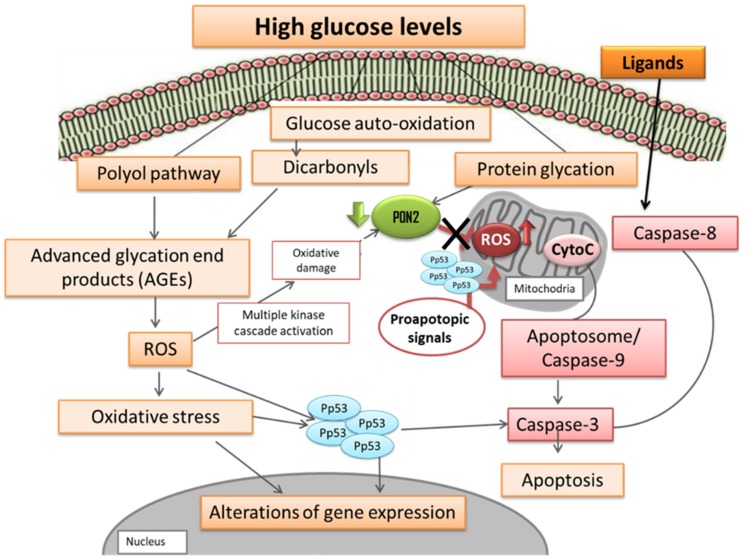
Biochemical pathways and molecular mechanisms of high glucose toxicity in Caco-2 cells and potential PON2 involvement in intracellular ROS formation and apoptosis. High glucose concentration can promote ROS accumulation through different metabolic pathways: glucose autoxidation, increased flux of glucose through the polyol pathway, AGEs formation, and decrease of cell antioxidant defenses. The decrease of PON2 expression and activity induced by glyco-oxidative stress may contribute to the intracellular ROS formation, cytochrome c release, and caspase activation and consequent activation of the mitochondrial pathway of apoptosis.

Figure 8 summarizes some biochemical pathways and potential mechanisms of action for glucose toxicity and involvement of PON2 in intracellular ROS formation and apoptosis in HG-treated Caco-2 cells.

Until today, the effect of high glucose levels on oxidative stress in intestinal cells has not been investigated. In regards to the physio-pathological consequences of our results, we suggest that the higher oxidative stress in intestinal cells can lead to mucosal barrier damage, which could allow pathogen bacteria to invade the submucosa and initiate an immune cascade. In fact, Draganov et al. [65] have shown that PON2 lactonase activity may have a role in disrupting quorum sensing by pathogenic bacteria. Quorum sensing is a signaling mechanism used by both gram-positive and gram-negative bacteria. Our hypothesis is supported by literature data on the alterations of intestinal permeability in high glucose-treated intestinal cells. In vitro studies using Caco-2 cells treated with too high glucose concentrations (50 mM) demonstrated alterations of tight junction integrity evaluated by analysis of zonula occludens-1 (ZO-1) [16]. Using intestinal epithelial cell (IEC-6) lower transepithelial/transendothelial electrical resistance (TEER) values, damage to the barrier structure and function have been reported in cells treated with a high concentration of glucose (50 mM) [66]. Moreover, alterations of intestinal glucose transport and epithelial integrity leading to an abnormal influx of immune stimulatory microbial products and a propensity for systemic spread of entry of pathogens has been demonstrated in animal models [16,67,68].

In conclusion, our results could be useful to understand the metabolic alterations due to high glycemic index diets and suggest that downregulation of PON2 after exposure to high glucose could be involved in intestinal cell dysfunction and increased risk for infection.
